# Obesity and accumulation of subcutaneous adipose tissue are poor prognostic factors in patients with alcoholic liver cirrhosis

**DOI:** 10.1371/journal.pone.0242582

**Published:** 2020-11-17

**Authors:** Akira Sakamaki, Kunihiko Yokoyama, Kyutaro Koyama, Shinichi Morita, Hiroyuki Abe, Kenya Kamimura, Masaaki Takamura, Shuji Terai

**Affiliations:** Division of Gastroenterology and Hepatology, Graduate School of Medical and Dental Sciences, Niigata University, Niigata, Japan; University of Navarra School of Medicine and Center for Applied Medical Research (CIMA), SPAIN

## Abstract

In alcoholic liver cirrhosis (LC) patients, obesity has become a problem that progresses into liver dysfunction. Herein, we investigated the relationship between the prognosis of steatohepatitis and body weight, along with fat accumulation in patients with alcoholic LC. We conducted a single-center retrospective study, enrolled 104 alcoholic LC patients without hepatocellular carcinoma (HCC) based on histological and clinical evidence, and investigated factors related to poor prognosis using multivariate Cox regression and cluster analyses. Cox regression analysis revealed three independent relevant factors: subcutaneous adipose tissue (SAT) index (median 34.8 cm^2^/m^2^, P = 0.009, hazard ratio [HR] 1.017, 95% confidence interval [CI] 1.004–1.030), total bilirubin level (median 1.7 mg/dL, P = 0.003, HR 1.129, 95% CI 1.042–1.223), and prothrombin time value (median 64%, P = 0.007, HR 0.967, 95% CI 0.943–0.991). In the cluster analysis, we categorized the patients into three groups: no adipose tissue accumulation (NAT group), SAT prior accumulation (SAT group), and visceral adipose tissue prior accumulation (VAT group). The results of the three groups revealed that the SAT group displayed a significantly poor prognosis of the Kaplan–Meier curve (67.1 vs 21.2 vs 65.3, P<0.001) of a 5-year survival rate. Propensity score matching analysis of the SAT and VAT groups was performed to adjust the patient’s background, but no significant differences were found between them; however, the prognosis was poorer (21.2 vs 66.3, P<0.001), and hemostatic factors were still at a lower level in the SAT group. These findings suggest that SAT accumulation type of obesity is a poor prognostic factor in alcoholic LC patients without HCC, and the hemorrhagic tendency might worsen the poor prognosis in such cases.

## Introduction

Because of the development of antiviral agents with negligible adverse effects switched from interferon therapies, liver diseases caused by the hepatitis C virus have decreased gradually and undoubtedly [1]. Therefore, steatohepatitis caused by alcoholic and nonalcoholic has become a significant issue of concern even with the decrease in the commonly known viral hepatitis as the causal agent [2]. In practice, a nationwide survey for hepatocellular carcinoma (HCC) in Japan by Tateishi et al. indicated an increased etiology rate of non-B, non-C hepatitis, while there was a decrease in the incidence of the hepatitis C virus [3].

The distinction between alcoholic and nonalcoholic liver diseases is often difficult, owing to the unreliability of alcohol consumption history [4]. When chronic inflammation and fibrosis of the liver were induced by nutritional disorder, oxidative stress, inflammatory cytokines, adipocytokines, and dysbiosis were observed in both alcoholic and nonalcoholic steatohepatitis [5, 6]. In the pathogenic mechanisms of alcoholic liver injury, the biological metabolic changes and acetaldehyde metabolized by alcohol-induced injury [5], in particular, by dysbiosis and endotoxin via direct injury of gut mucosa by the alcohol, and are also notable factors of liver damage [7].

Previous studies showed that nutritional disorder that focused on malnutrition and vitamin deficiency accounted for patients with alcoholic liver cirrhosis (LC), and heavy drinking, irregular feeding habits, and decompensated cirrhosis with ascites are involved in malnutrition [8]. In fact, a previous report indicated that subcutaneous adipose tissue (SAT) index in females and skeletal muscle mass index (SMI) in males were significant predictors of mortality in patients with cirrhosis of all backgrounds [9]. Controversially, the guideline of the European Association for the Study of the Liver for alcohol-related liver disease showed that obesity is the risk factor for liver fibrosis and cirrhosis in patients with alcoholic LC [10]. Now, obesity and glucose intolerance have become problems of nutritional disorders, and hypernutrition could also progress into liver dysfunction in alcohol processing [11].

Herein, we investigated and reported the relationship between prognosis and body weight, as well as fat accumulation in patients with alcoholic LC.

## Materials and methods

### Data collection and inclusion and exclusion criteria

This retrospective study was performed at Niigata University Hospital. The study was approved by the ethical review board of Niigata University (Approval Number 2019–0225).

Data were collected from hospital medical records of patients diagnosed with LC between January 2006 and December 2019. LC diagnosis was based on histological and clinical evidence. Clinical diagnosis of LC was based on a previous report [12], as follows: (1) platelet count of <100,000/μL and ultrasonography findings suggestive of cirrhosis and (2) clinical signs of portal hypertension, such as ascites, esophageal or gastric varices, and hepatic encephalopathy. A total of 687 cases met with the criteria for LC. Of these patients, alcohol-induced LC was diagnosed based on past and/or current history of alcohol abuse (over 60g/day) by self-report without the complication of other reason of liver injuries, such as viral or autoimmune hepatitis, Wilson’s disease, or idiopathic portal hypertension. Among the 201 who satisfied the inclusion criteria, we excluded 20 patients wish insufficient data to analyze their liver function, physical examinations, and body composition, and 77 patients with complications of HCC. Patients with a history of HCC were included if they showed no recurrence for more than 3 years after the last treatment. The remaining 104 patients were assessed in the final analyses. The number of cases in the hospital during the study period determined the sample size. The primary study endpoint was set as overall survival, and the secondary study endpoint was set as the incidence of a new occurrence of HCC.

### Assessment of body composition by computed tomography (CT) scan

Because of Niigata University Hospital is the higher-level functional hospital in Niigata prefecture, Japan, patients were referred from other hospitals for professional treatment, such as endoscopic submucosal dissection for esophageal cancer, endoscopic injection sclerotherapy or variceal ligation, or balloon-occluded retrograde transvenous obliteration of gastroesophageal varices, and surgery or transcatheter arterial chemoembolization for HCC (HCC cases were excluded in this study). Therefore, we performed CT in almost all cases (approximately 90%) to determine the therapeutic strategy for these problems.

A transverse CT image from the third lumbar vertebra was identified from each retrieved scan. The CT images were analyzed using hand segmentation with the help of image analysis software SliceOmatic version 5.0 (TomoVision, Magog, QC, Canada).

The identification of skeletal muscle and adipose tissue was based on predefined CT density of −29 to +150 [13] and −190 to −30 Hounsfield Unit [14], respectively. Skeletal muscle and adipose tissue area were then normalized for stature (height in m^2^) and expressed as SMI, SAT index, and visceral adipose tissue (VAT) index in cm^2^/m^2^. The total adipose tissue (TAT) was determined by the total of the SAT and VAT. Besides, TAT was also normalized for stature (TAT index, cm^2^/m^2^).

### Statistical analysis

Cox regression and Kaplan–Meier analysis were used to compare the prognosis, and the cumulative incidence plots method was used to compare the plausible incidence of HCC. The Kolmogorov–Smirnov test was used to assess the normality of the distribution of continuous variables. Spearman’s rank coefficient of correlation was used to evaluate correlativity. Mann–Whitney U, Kruskal–Wallis, and Fisher’s exact tests were used to compare data of each cluster. The propensity score matching analysis was used to adjust the factors of SAT and VAT patient groups. Propensity score was calculated using the adjusting factors: age, body mass index (BMI) and total bilirubin level. Cases in the VAT group were selected that corresponded to those in the SAT group by the nearest neighbor method. SPSS Statistics software (version 22.0; IBM, Armonk, NY, USA) was used to perform Spearman’s rank coefficient of correlation, the Kolmogorov–Smirnov, Mann–Whitney U, Kruskal–Wallis, and Fisher’s exact tests, and propensity score matching, whereas the Prism Software (version 8.30; GraphPad, La Jolla, CA, USA) was used to perform Kaplan–Meier and the cumulative incidence plots.

## Results

**Fig 1:**
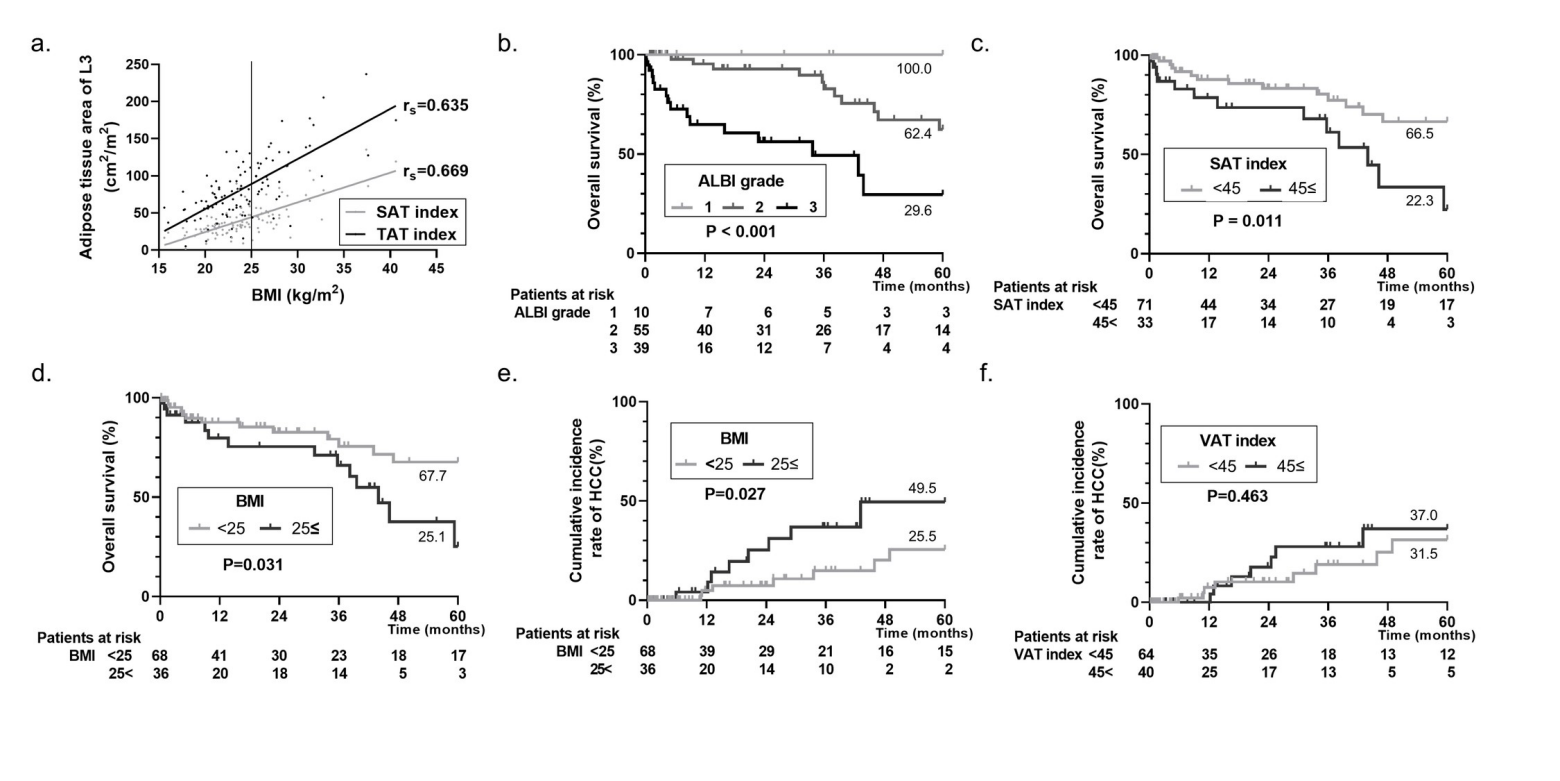
Kaplan–Meier curve and cumulative incidence plot based on the univariate Cox regression analysis. BMI was correlated with the adipose tissue area (a): SAT index (r_s_ = 0.669) and TAT index (r_s_ = 0.635). 45 cm^2^/m^2^ of SAT index and 90 cm^2^/m^2^ of TAT index were equivalent to 25 kg/cm^2^ of BMI. The Kaplan–Meier curve indicated significantly poor prognosis in an advanced grade of liver function on ALBI grade (P < 0.001, 5-year survival rates were 100.0 vs 62.4 vs 29.6, b) and the accumulation of SAT (P = 0.011, HR 3.931, 95% CI 1.286–7.142, the cutoff value of SAT index was 45 cm^2^/m^2^, 5-year survival rates were 66.5 vs 22.3, c). Furthermore, obesity was also revealed as a significantly poor prognosis (P = 0.031, HR 2.440, 95% CI 1.084–5.490, the cutoff value of BMI was 25 kg/cm^2^, 5-year survival rates were 67.7 vs 25.1, d) but not more marked than SAT. The cumulative incidence rate indicated significantly higher HCC complication in obesity (P = 0.027, HR 3.652, 95% CI 1.158–11.52, the cutoff value of BMI was 25 kg/cm^2^, 5-year incidence rate were 25.5 vs 49.5, e), but no differences in the accumulation of VAT (P = 0.463, HR 1.482, 95% CI 0.518–4.243, the cutoff value of VAT index was 45 cm^2^/m^2^, 5-year incidence rate were 31.5 vs 37.0, f). L3, third lumbar vertebra; BMI, body mass index; SAT, subcutaneous adipose tissue; TAT, total adipose tissue; ALBI, albumin–bilirubin; HCC, hepatocellular carcinoma; VAT, visceral adipose tissue; CI, confidence interval.

The study cohort included 82 males and 22 females (n = 104), with a median age of 60 years. The median observation period in the entire cohort was 1.6 years. Furthermore, 16 patients (15.4%) had HCC as a complication, and 30 (28.8%) died during the study period. The median score of the Charlson comorbidity index [15] was 4. BMI correlated with the adipose tissue area: SAT index (r_s_ = 0.669), VAT index (r_s_ = 0.484), and TAT index (r_s_ = 0.635). 45 cm^2^/m^2^ of the SAT and VAT indexes and 90 cm^2^/m^2^ of the TAT index were equivalent to 25 kg/m^2^ of BMI, and 55 cm^2^/m^2^ of the TAT index was equivalent to 20 kg/m^2^ of BMI in the correlation analysis (Fig 1a).

First, we investigated the relevant factors for prognosis in 104 patients with alcoholic LC by univariate Cox regression analysis (Table 1) and found nine, as follows: BMI, SAT index, aspartate aminotransferase, alanine aminotransferase, albumin, total bilirubin, prothrombin time, fibrinogen and platelet count. Furthermore, multivariate Cox regression analysis revealed three independent factors: SAT index (P = 0.009, hazard ratio [HR] 1.017, 95% confidence interval [CI] 1.004–1.030), total bilirubin (P = 0.003, HR 1.129, 95% CI 1.042–1.223), and prothrombin time (P = 0.007, HR 0.967, 95% CI 0.943–0.991). That is the accumulation of SAT and liver dysfunction correlated with the poor prognosis in patients with alcoholic LC. The Kaplan–Meier curve indicated significantly poor prognosis in an advanced grade of liver function on albumin–bilirubin (ALBI) grade [16] (P < 0.001, 5-year survival rates were 100.0 vs 62.4 vs 29.6, Fig 1B) and the accumulation of SAT (P = 0.011, HR 3.931, 95% CI 1.286–7.142, the cutoff value of SAT index was 45 cm^2^/m^2^, 5-year survival rates were 66.5 vs 22.3, Fig 1C). Furthermore, obesity was also a significantly poor prognosis (P = 0.031, HR 2.440, 95% CI 1.084–5.490, the cutoff value of BMI was 25 kg/cm^2^, 5-year survival rates were 67.7 vs 25.1, Fig 1D) but not more marked than SAT.

**Table 1:** Cox regression for prognosis and hepatic carcinogenesis in patients with alcoholic liver cirrhosis.

	N = 104	P value		P value	
Cox regression	median (min–max)	Prognosis		Hepatic carcinogenesis	
(Univariate or Multivariate)	or n (%)	Univariate	Multivariate	Univariate	Multivariate
**Age, years**	60 (30–82)	0.827		0.054	
**Gender**					
Males	83 (79.8)	0.898		0.074	
Females	21 (20.2)				
**Gastroesophageal varices**	18 / 20 / 48 / 15	0.340		0.680	
(F0 / F1 / F2 / F3 or rupture)					
**Ascites**	59 / 23 / 22	0.051		0.286	
(None / mild / moderate to severe)					
**Overt hepatic encephalopathy**	5 (4.8)	0.514		0.258	
**Charlson comorbidity index**	4 (3–7)	0.063		0.043*	0.045*
**Body mass index, kg/m**^**2**^	23.8 (15.6–40.6)	0.011*	0.713	0.028*	0.030*
**Skeletal muscle mass index, cm**^**2**^**/m**^**2**^	43.6 (21.5–71.9)	0.802		0.206	
**SAT index, cm**^**2**^**/m**^**2**^	34.8 (1.8–135.3)	0.013*	0.009*	0.163	
**VAT index, cm**^**2**^**/m**^**2**^	39.3 (3.3–125.0)	0.687		0.032*	0.598
**TAT index, cm**^**2**^**/m**^**2**^	76.1 (5.1–236.9)	0.141		0.055	
**Aspartate aminotransferase, U/L**	50 (15–538)	0.023*	0.094	0.145	
**Alanine aminotransferase, U/L**	29 (10–576)	0.026*	0.474	0.340	
**Albumin, g/dL**	3.1 (1.5–4.9)	0.004*	0.806	0.946	
**Total bilirubin, mg/dL**	1.7 (0.5–27.1)	<0.001*	0.003*	0.278	
**Gamma-glutamyl transpeptidase, U/L**	117 (15–1167)	0.772		0.358	
**Cholinesterase, U/L**	122 (30–359)	0.056		0.747	
**Prothrombin time, %**	64 (17–110)	<0.001*	0.007*	0.688	
**Fibrinogen, mg/dL**	191 (43–581)	0.006*	0.453	0.131	
**Ammonia, μg/dL**	95 (25–311)	0.185		0.099	
**Creatinine, mg/dL**	0.74 (0.36–2.95)	0.236		0.591	
**Blood urea nitrogen, mg/dL**	13 (3–84)	0.350		0.313	
**eGFR, mL/min/1.73m**^**2**^	81.3 (14.0–159.0)	0.542		0.599	
**White blood cell count, x10**^**3**^**/μL**	5.31 (39.88–2.45)	0.061		0.102	
**Platelet count, x10**^**4**^**/μL**	9.3 (3.3–31.8)	0.020*	0.142	0.293	
**C-reactive protein, mg/dL**	0.28 (0.01–17.47)	0.861		0.218	
**Hemoglobin A1c, %**	5.2 (3.2–9.4)	0.128		0.376	
**Post definitive therapy for HCC**	4 (3.8)	0.356		0.419	
**Child-Pugh score**	8 (5–13)	0.009*		0.221	
**ALBI score**	-1.67 (-3.32–0.30)	<0.001*		0.851	
**5-year incidence rate of HCC**	34.7	-		-	
**5-year survival rate**	54.0	-		-	

SAT, subcutaneous adipose tissue; VAT, visceral adipose tissue; TAT, total adipose tissue; eGFR, estimated glomerular filtration rate; HCC, hepatocellular carcinoma

*:P value < 0.05.

We then investigated the relevant factors for the incidence of HCC by univariate Cox regression analysis and found three factors (Charlson comorbidity index, BMI, and VAT index), and multivariate Cox regression analysis revealed only two factors, Charlson comorbidity index (P = 0.045, HR 1.587, 95% CI 1.011–2.493), and BMI (P = 0.028, HR 1.121, 95% CI 1.012–1.241), which remained significantly different (Table 1). The cumulative incidence rate indicated significantly higher HCC complication in obesity (P = 0.027, HR 3.652, CI 1.158–11.52, the cutoff value of BMI was 25 kg/cm^2^, 5-year incidence rates were 25.5 vs 49.5, Fig 1E), but there were no differences in the accumulation of VAT (P = 0.463, HR 1.482, CI 0.518–4.243, the cutoff value of VAT index was 45 cm^2^/m^2^, 5-year incidence rate were 31.5 vs 37.0, Fig 1F). The results showed that obesity and the accumulation of adipose tissue had a strong influence on the prognosis and carcinogenesis of alcoholic LC, especially since the SAT is related to the prognosis. Cutoff values of SAT and VAT indexes were defined as the same method of previous report [17] by using the results of the correlation analysis (Fig 1A) that were equivalent to 25 kg/m^2^ of BMI. In addition, one-year survival rate was 87.1, and 3-year survival rate was 46.4 after the incidence of HCC (S1 Fig).

**Fig 2:**
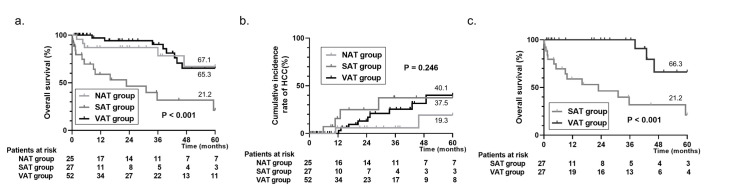
Kaplan–Meier curve and cumulative incidence plot based on the cluster analysis. We provided three groups: No accumulation of adipose tissue (TAT index <55 cm^2^/m^2^, NAT group), SAT prior accumulation (TAT index ≥ 55 cm^2^/m^2^ and SAT>VAT, SAT group), and VAT prior accumulation (TAT index ≥ 55 cm^2^/m^2^ and VAT>SAT, VAT group) in the cluster analysis. As the result of a comparison of the three groups, the SAT group was a significantly poor prognosis in the Kaplan–Meier curve (P < 0.001, 5-year survival rates were 67.1 vs 21.2 vs 65.3, a), and there was no difference in the incidence of HCC in the cumulative incidence plots (P = 0.246, 5-year incidence rates were 19.3 vs 37.5 vs 40.1, b). Furthermore, we compared the SAT and VAT groups using propensity score matching analysis after adjusting for three factors: Age, body mass index, and serum total bilirubin level. Twenty-seven SAT, and VAT group patients were 1:1 matched, and the Kaplan–Meier curve indicated the prognosis was poorer (P < 0.001, HR 6.154, 95% CI 2.306–16.42, 5-year survival rates were 21.2 vs 66.3, c) among the SAT group than the VAT group. TAT, total adipose tissue; SAT, subcutaneous adipose tissue; VAT, visceral adipose tissue; HCC, hepatocellular carcinoma; CI, confidence interval.

To further analyze the difference of distribution of adipose tissue, we provided the following three groups: no accumulation of adipose tissue (TAT index < 55 cm^2^/m^2^, NAT group), SAT prior accumulation (TAT index ≥ 55 cm^2^/m^2^ and SAT > VAT, SAT group), and VAT prior accumulation (TAT index ≥ 55 cm^2^/m^2^ and VAT > SAT, VAT group) as the cluster analysis (Table 2). As a result, comparison of the three groups was made; the SAT group was a significantly poor prognosis in the Kaplan–Meier curve (P < 0.001, 5-year survival rates were 67.1 vs 21.2 vs 65.3, Fig 2A) and no difference in the incidence of HCC in the cumulative incidence plots (P = 0.246, 5-year incidence rates were 19.3 vs 37.5 vs 40.1, Fig 2B). To reveal the relationship between SAT accumulation and prognosis, we investigated the cause of death and revealed significant differences among the three groups (Table 2, P = 0.039). In detail, acute and chronic liver failure and malignancy, including HCC, showed no significant difference (P = 0.664 and 1.000), while bleeding was higher in the SAT group (P = 0.038), and infection was higher in the VAT group (P = 0.019). Furthermore, all of non-variceal bleeding was found in the SAT group; specifically, muscle hepatoma was 75.0% (4/6) of the non-variceal bleeding.

**Table 2:** Cluster analysis based on the accumulation type of adipose tissue.

	NAT group	SAT group	VAT group		
	N = 25	N = 27	N = 52	P value	
Kruskal–Wallis, Mann–Whitney U	median (min–max)			NAT vs SAT	
or Fisher’s exact tests	or n (%)			vs VAT	SAT vs VAT
**Age, years**	49 (33–71)	57 (39–74)	64 (33–82)	0.001*	0.025*
**Gender**					
Males	18 (72.0)	20 (74.1)	45 (86.5)	0.225	0.217
Females	7 (28.0)	7 (25.9)	7 (13.5)		
**Gastroesophageal varices**	6 / 5 / 11 / 3	4 / 6 / 10 / 4	8 / 9 / 27 / 8	0.930	0.785
(F0 / F1 / F2 / F3 or rupture)					
**Ascites**	10 / 7 / 8	16 / 4 / 7	33 / 12 / 7	0.190	0.372
(None / mild / moderate to severe)					
**Overt hepatic encephalopathy**	0 (0.0)	3 (11.1)	2 (3.8)	0.225	0.331
**Charlson comorbidity index**	3 (3–6)	4 (4–7)	4 (4–7)	0.656	0.553
**Body mass index, kg/m**^**2**^	21.9 (16.0–29.2)	26.1 (20.3–40.6)	23.6 (15.6–32.8)	<0.001*	0.005*
**Skeletal muscle mass index, cm**^**2**^**/m**^**2**^	42.4 (28.0–65.3)	44.4 (23.1–59.6)	43.6 (21.5–71.9)	0.564	0.549
**SAT index, cm**^**2**^**/m**^**2**^	20.4 (1.8–33.4)	52.3 (31.8–135.3)	35.9 (16.7–80.5)	<0.001*	<0.001*
**VAT index, cm**^**2**^**/m**^**2**^	15.0 (3.3–38.3)	36.1 (14.5–101.6)	48.9 (28.1–125.0)	<0.001*	0.002*
**TAT index, cm**^**2**^**/m**^**2**^	39.9 (5.0–54.8)	86.6 (58.4–236.9)	83.5 (56.0–205.5)	<0.001*	0.352
**Aspartate aminotransferase, U/L**	65 (17–301)	51 (18–538)	43 (15–326)	0.284	0.570
**Alanine aminotransferase, U/L**	32 (14–59)	33 (12–576)	28 (10–83)	0.540	0.466
**Albumin, g/dL**	3.4 (1.5–4.4)	3.1 (1.7–4.5)	3.1 (1.7–4.9)	0.390	0.185
**Total bilirubin, mg/dL**	2.1 (0.5–27.1)	2.3 (0.6–23.4)	1.3 (0.5–8.2)	0.013*	0.006*
**Gamma-glutamyl transpeptidase, U/L**	86 (19–1167)	70 (15–729)	154 (15–923)	0.035*	0.015*
**Cholinesterase, U/L**	123 (39–269)	124 (30–275)	118 (39–359)	0.935	0.636
**Prothrombin time, %**	64 (22–110)	48 (17–89)	72 (35–108)	0.003*	0.001*
**Fibrinogen, mg/dL**	192 (46–482)	163 (43–348)	220 (74–581)	0.011*	0.002*
**Ammonia, μg/dL**	86 (39–150)	116 (32–311)	87 (25–261)	0.284	0.160
**Creatinine, mg/dL**	0.61 (0.40–2.02)	0.70 (0.36–2.95)	0.83 (0.42–2.80)	0.010*	0.118
**Blood urea nitrogen, mg/dL**	11 (3–47)	13 (4–84)	14 (4–55)	0.060	0.776
**eGFR, mL/min/1.73m**^**2**^	93.8 (23.7–159.0)	87.6 (14.0–142.6)	71.5 (18.7–143.7)	0.016*	0.235
**White blood cell count, x10**^**3**^**/μL**	4.5 (2.7–18.9)	6.4 (2.6–39.9)	5.3 (2.5–29.0)	0.327	0.235
**Platelet count, x10**^**4**^**/μL**	9.9 (3.7–18.4)	6.8 (3.3–31.8)	9.8 (4.4–23.0)	0.079	0.025*
**C-reactive protein, mg/dL**	0.24 (0.01–16.27)	0.33 (0.01–11.05)	0.29 (0.03–17.47)	0.726	0.679
**Hemoglobin A1c, %**	4.8 (3.2–7.0)	5.3 (3.6–8.6)	5.4 (4.1–9.4)	0.038*	0.813
**Post definitive therapy for HCC**	1 (4.0)	0 (0.0)	3 (5.8)	0.676	0.547
**Child-Pugh score**	8 (5–13)	9 (5–13)	7 (5–13)	0.083	0.038*
**ALBI score**	-1.74(-3.03–0.30)	-1.57 (-2.91–0.22)	-1.72 (-3.32- -0.32)	0.186	0.051
**5-year incidence rate of HCC**	19.3	37.5	40.1	0.246	0.462
**5-year survival rate**	67.1	21.2	65.3	<0.001*	<0.001*
**Fatal cases**	6 (24.0)	14 (51.9)	10 (19.2)	0.012*	0.004*
**Cause of death**				0.039*	0.010*
Liver failure, all (acute on chronic)	3 (0)	4 (1)	3 (0)		
Malignancy, all organs (HCC)	1 (0)	2 (1)	1 (0)		
Bleeding, all (variceal, muscle hematoma, others)	2 (2,0,0)	8 (2,4,2)	0 (0,0,0)		
General infection	0	0	3		
Others / Unspecified	0	0	3		

SAT, subcutaneous adipose tissue; VAT, visceral adipose tissue; TAT, total adipose tissue; eGFR, estimated glomerular filtration rate; HCC, hepatocellular carcinoma; ALBI, albumin-bilirubin

*:P value < 0.05.

A direct comparison of SAT and VAT groups showed statistical differences in the following: age (younger in SAT, P = 0.025), BMI (higher in SAT, P = 0.005), total bilirubin (higher in SAT, P = 0.006), and prothrombin time (lower in SAT, P = 0.001), fibrinogen (lower in SAT, P = 0.002), and platelet count (lower in SAT, P = 0.025). Therefore, we compared two groups using propensity score matching analysis after adjusting for the three factors, age, BMI, and serum total bilirubin level. Twenty-seven SAT, and VAT patient groups were 1:1 matched, and no significant differences were found between the two groups in terms of age, gender, BMI, and the ALBI grade (Table 3). The prognosis was poorer (P < 0.001, HR 6.154, 95% CI 2.306–16.42, 5-year survival rates were 21.2 vs 66.3, Fig 2C), and prothrombin time (P = 0.006), fibrinogen (P = 0.015), and platelet count (P = 0.033) were still at the lower levels (Table 3) among the SAT group than the VAT group.

**Table 3:** Propensity score matching analysis based on the accumulation type of adipose tissue.

	SAT group	VAT group	
	N = 27	N = 27	
Mann–Whitney U	median (min–max)		
or Fisher’s exact tests	or n (%)		P value
**Age, years**	57 (39–74)	60 (33–74)	0.527
**Gender**			
Males	20 (74.1)	23 (85.2)	0.501
Females	7 (25.9)	4 (14.8)	
**Gastroesophageal varices**	4 / 6 / 10 / 4	4 / 5 / 15 / 3	0.797
(F0 / F1 / F2 / F3 or rupture)			
**Ascites**	16 / 4 / 7	16 / 7 / 4	0.419
(None / mild / moderate to severe)			
**Overt hepatic encephalopathy**	3 (11.1)	2 (7.4)	1.000
**Charlson comorbidity index**	4 (4–7)	4 (4–7)	1.000
**Body mass index, kg/m**^**2**^	26.1 (20.3–40.6)	25.7 (15.6–32.8)	0.333
**Skeletal muscle mass index, cm**^**2**^**/m**^**2**^	44.4 (23.1–59.6)	45.5 (21.5–71.9)	0.634
**SAT index, cm**^**2**^**/m**^**2**^	52.3 (31.8–135.3)	37.1 (16.7–80.5)	0.001*
**VAT index, cm**^**2**^**/m**^**2**^	36.1 (14.5–101.6)	49.4 (34.7–125.0)	0.002*
**TAT index, cm**^**2**^**/m**^**2**^	86.6 (58.4–236.9)	91.8 (57.1–205.5)	0.697
**Aspartate aminotransferase, U/L**	51 (18–538)	43 (20–138)	0.762
**Alanine aminotransferase, U/L**	33 (12–576)	29 (12–69)	0.822
**Albumin, g/dL**	3.1 (1.7–4.5)	3.1 (1.8–4.9)	0.264
**Total bilirubin, mg/dL**	2.3 (0.6–23.4)	1.3 (0.6–8.2)	0.093
**Gamma-glutamyl transpeptidase, U/L**	70 (15–729)	243 (37–706)	0.005*
**Cholinesterase, U/L**	124 (30–275)	122 (39–359)	0.460
**Prothrombin time, %**	48 (17–89)	71 (35–108)	0.006*
**Fibrinogen, mg/dL**	163 (43–348)	210 (81–464)	0.015*
**Ammonia, μg/dL**	116 (32–311)	87 (25–261)	0.149
**Creatinine, mg/dL**	0.70 (0.36–2.95)	0.82 (0.42–1.92)	0.216
**Blood urea nitrogen, mg/dL**	13 (4–84)	13 (7–55)	0.910
**eGFR, mL/min/1.73m**^**2**^	87.6 (14.0–142.6)	72.8 (26.6–143.7)	0.505
**White blood cell count, x10**^**3**^**/μL**	6.4 (2.6–39.9)	5.8 (2.5–29.0)	0.447
**Platelet count, x10**^**4**^**/μL**	6.8 (3.3–31.8)	9.2 (6.0–23.0)	0.033*
**C-reactive protein, mg/dL**	0.33 (0.01–11.05)	0.33 (0.04–15.24)	0.890
**Hemoglobin A1c, %**	5.3 (3.6–8.6)	5.4 (4.6–8.3)	0.693
**Post definitive therapy for HCC**	0 (0.0)	0 (0.0)	1.000
**Child-Pugh Score**	9 (5–13)	7 (5–13)	0.135
**ALBI score**	-1.57 (-2.91–0.22)	-1.68 (-3.32- -0.37)	0.161
**5-year incidence rate of HCC**	37.5	38.9	0.593
**5-year survival rate**	21.2	66.3	<0.001*

SAT, subcutaneous adipose tissue; VAT, visceral adipose tissue; TAT, total adipose tissue; eGFR, estimated glomerular filtration rate; HCC, hepatocellular carcinoma; ALBI, albumin-bilirubin

*:P value < 0.05.

## Discussion

Obesity is the risk factor for alcoholic LC as previously mentioned [10]. The evidence is based on cross-sectional studies [11, 18], which did not observe each case and assess the prognosis. The relationship between obesity and hepatocarcinogenesis was analyzed by the same method [19]. Our follow-up study of alcoholic LC also indicated obesity was the risk factor of the poor prognosis and HCC. Previous reports indicated that the incidence of HCC in patients with alcoholic LC was 2.6% to 2.9% a year [20, 21], although this study found a greater incidence of HCC (approximately 6.9% a year). These reports indicated that the risk factors for HCC incidence was age and platelet count. Mancebo et al. [20] revealed that older patients (≥55 years old) and those with a low platelet count (<125,000/mm^3^) were at greater risk for HCC (4.8% a year). In our study, the median age was 60 and median platelet count was 93,000/mm^3^, which indicated that patients at high risk for HCC were included more than in previous studies. Furthermore, Ganne-Carrié et al. [21] indicated a poor prognosis after HCC diagnosis (1-year survival, 64.3%). In this study, HCC complication was a good prognosis suggesting the earlier detection of HCC than in previous reports.

Since the condition of many LC patients were complicated with ascites that might cause inaccuracy of the BMI measurements, we used L3-level adipose accumulation in CT scan to exclude the influence of ascites and revealed SAT accumulation was more marked than BMI for prognosis in alcoholic patients with LC.

It is well known that VAT accumulation generates a harmful effect on the human body. A large clinical study in Japan revealed that the mean number of obesity-related cardiovascular risk factors (hypertension, low high-density lipoprotein cholesterolemia and/or hypertriglyceridemia, and abnormal glucose levels) significantly correlated with the VAT area but not the SAT area [22]. In the field of hepatic disease, VAT affects the progression of the pathological condition in patients with nonalcoholic fatty liver disease [23]. Therefore, visceral fat type obesity was defined over 0.4 of visceral to subcutaneous adipose tissue ratio [24], or over 100 cm^2^ of VAT area [17] by the measurement of CT images. On the other hand, subcutaneous fat type obesity was not clearly defined.

There is no precise elucidation regarding the difference in the distribution and function between SAT and VAT; however, it is known that the SAT tends to exhibit differential proliferation but not VAT [25]. For hypernutritional load, the human body responded through cell proliferation in subcutaneous adipose cells, and cell enlargement in visceral adipose cells was observed. However, adipose cell proliferation is found in only childhood and pregnancy and childbearing period in females [26]. That is, for hypernutrition after adulthood, the human body responded through cell enlargement in visceral adipose cells, and overloaded nutrient contents that could not be handled the cell enlargement in VAT stored in the liver, skeletal, or cardinal muscle, and it is called ectopic fat deposition. The enlarged visceral fat cells assume a hypoxic state, triggering induced chronic inflammation by apoptosis, infiltration of immune cells, and abnormal functions of adipose cells, such as an increase of oxidation stress, and the abnormal production of adipocytokines [27]. Therefore, VAT accumulation might induce and worsen the general inflammation, that compatible with our results that showed the relationship between VAT type obesity and infection-related death.

Although clinical results and mechanisms have been established previously, our data revealed that the accumulation of SAT significantly correlated with poor prognosis. According to our analysis of the cause of the death in alcoholic LC, a significantly high incidence of fatal bleeding complications was found, primarily, non-variceal bleeding, as typified by muscle hematoma. On the other hand, no significant difference between liver failure and HCC was noted. Alcoholic LC had a higher incidence of non-variceal bleedings than other occlusions of cirrhosis [28], and there have been reports on the inhibition of platelet adhesion to fibrinogen [29], suppression of platelet aggregation by ethanol [30], and the promotion of atherosclerosis [31] as the mechanism of the hemorrhagic tendency in patients with alcoholic LC [32]. Furthermore, muscle hematoma is one of the poor prognostic complications in patients with LC, and almost all of the complications are detected in alcoholic LC. A literature review of eight cases of muscle hematoma in patients with LC indicated that 87.5% (7/8) were caused by alcohol, and 75.0% (6/8) were by fatal events [33]. For a more precise comparison, we matched the background of the accumulation of SAT and VAT by propensity matching analysis and revealed poor prognosis of the SAT group. Interestingly, no significant differences in ALBI scores were found between the two groups after adjusting, hemostatic, and coagulation factors were still at a lower level in the SAT group. This result might reflect the hemorrhagic tendency in alcoholic LC patients with SAT accumulation.

Our present study has the following limitations. First, the amount of alcohol intake after the diagnosis of alcoholic LC could not be assessed because this is a retrospective study. It is well known that complete abstinence from alcohol could reduce the risk of liver-related complications and mortality [34, 35], and this factor might affect the results. To minimize the influence of such unvalued factors, we used two methods (Cox regression and propensity score matching analyses) to determine the relationship between the poor prognosis and the SAT accumulation, which yielded the same results. Second, we could not find the direct relationship and mechanism between the differences in adipose distribution and the hemorrhagic tendency in patients with alcoholic LC. Therefore, further analyses are needed to establish the relationship.

## Conclusions

SAT accumulation-type obesity in alcoholic LC patients without HCC is significantly related to poor prognosis. In this type, the complication of non-variceal bleeding, including muscle hematoma, characteristics in alcoholic LC was found. To follow these cases, we should pay more attention to the complication of HCC and bleeding. To predict the bleeding risk, we should confirm the hemostatic and coagulation factors and the adipose distribution.

## Supporting information

S1 Fig Kaplan–Meier curve after the incidence of hepatocellular carcinoma. One-year survival rate was 87.1 and 3-year survival rate was 46.4.
(TIF)
