# Supplementary figures and images for: Obesity and accumulation of subcutaneous adipose tissue are poor prognostic factors in patients with alcoholic liver cirrhosis
Source: PLoS One. 2020 Nov 17;15(11):e0242582. doi: 10.1371/journal.pone.0242582 (PMC7671528; doi:10.1371/journal.pone.0242582)

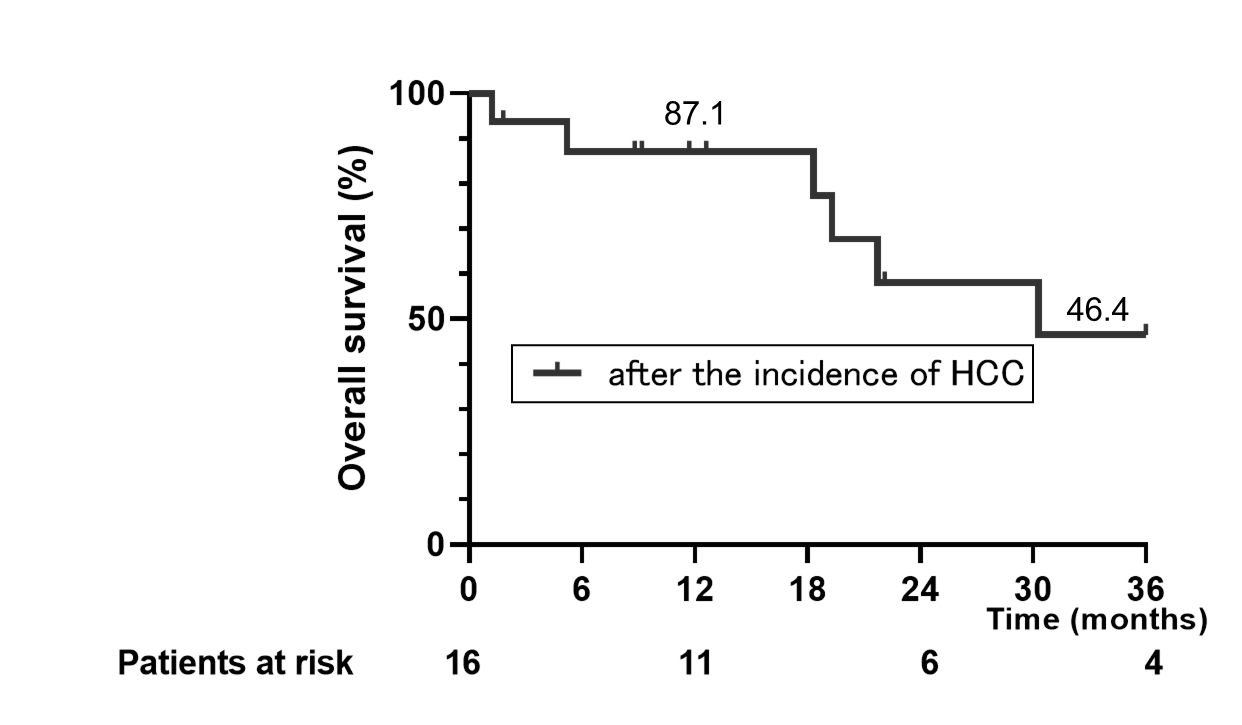

Supplement: S1 Fig — One-year survival rate was 87.1 and 3-year survival rate was 46.4. (TIF) [file pone.0242582.s001.TIF]
